# Web of lies: a tool for determining the limits of verification in preventing the spread of false information on networks

**DOI:** 10.1038/s41598-021-82844-7

**Published:** 2021-02-15

**Authors:** Kinga Makovi, Manuel Muñoz-Herrera

**Affiliations:** Social Science Division, New York University, Abu Dhabi, UAE

**Keywords:** Psychology, Human behaviour

## Abstract

The spread of false information on social networks has garnered substantial scientific and popular attention. To counteract this spread, verification of the truthfulness of information has been proposed as a key intervention. Using a novel behavioral experiment with over 2000 participants, we analyze participants’ willingness to spread false information in a network. All participants in the network have aligned incentives making lying attractive and countering the explicit norm of truth-telling that we impose. We investigate how verifying the truth, endogenously or exogenously, impacts the choice to lie or to adhere to the norm of truth-telling and how this compares to the spread of information in a setting in which such verification is not possible. The three key take-aways are (1) verification is only moderately effective in reducing the spread of lies, and (2) its effectivity is contingent on the agency of people in seeking the truth, and (3) on the exposure of liars, not only on the exposure of the lies being told. These results suggest that verification is not a blanket solution. To enhance its effectivity, verification should be combined with efforts to foster a culture of truth-seeking and with information on who is spreading lies.

## Introduction

The spread of false information on social networks has received a great deal of attention from both academic research and popular news^1–3^. This recent interest has been sparked by the alarming potential impact that false information may have had on election outcomes^4,5^. However, the concern is broader and extends to whether false information can influence support for specific policies^6^, whether or not one’s children should be vaccinated^7^ and if one should get a flu shot^8^. Against this backdrop, the study of interventions that can counteract the spread of false information on social networks is timely^9^. A widely proposed intervention to counteract the ills that false information may cause is promoting the verification of the information shared in networks^1^.

Verification can occur in two main ways: exogenously or endogenously. Exogenous verification is when an external and impartial source labels the veracity of information. For example, algorithms have been proposed to rank content by its credibility^10,11^. In this vein, Google has led an effort to rank search results by a trustworthiness score^12^. In other words, exogenous verification is a top-down solution. Endogenous verification is when those exchanging information take measures themselves to investigate the truth. For instance, Facebook spearheaded a controversial effort to crowd-source verification (https://www.facebook.com/zuck/videos/10106612617413491/). As such, endogenous verification is a bottom-up solution and depends heavily on the willingness of people to put effort towards truth seeking.

The underlying motivation behind verification is the presence of a widely held norm of truth-telling^13^. This means that when false information is identified, people can be expected to make that falsity known and not spread lies, even when it goes against their self-interest. Despite this normative expectation, the effectivity of verification may be compromised, as people do not act in a vacuum; rather, they act in a naturally occurring social network in which those connected to one another have similar dispositions, interests and incentives^14,15^. It has been shown that social networks have become more polarized over time^16–19^, which may lead people to prioritize fitting in and supporting views that are shared by other group members and thus beneficial to their group by reinforcing group identity^19–21^ instead of incorporating contradicting information^22,23^ and telling the truth^13,24^.

The tension between aligned interests and a wide social norm of truth-telling motivates our investigation on how well verification works and how its effectivity can be enhanced. Arguably, answering this question in a field setting is fraught with challenges for at least three reasons. First, it is nearly impossible to identify who verified the information shared, impeding the evaluation of how verification impacted the choice to spread false information. Second, even if tracking information on verification is possible, those who verify it may have different preferences for honesty than those who do not. As a consequence, it may be impossible to know if verification, if imposed, would be effective on those who do not typically verify information. Third, social connections are not random, and they may depend on preferences for honesty, as well as on the act of verification. In short, people are not randomized in their social positions, nor are they randomly exposed to verification regimes and their use.

To address these obstacles, we design and conduct an online experiment that provides us with a controlled environment where a verification regime can be randomly assigned and tracked. This allows us to observe which people know the truth about the information they spread and what type of verification is used to find the truth. Moreover, we have control over the social positions that people take, i.e., participants do not choose their interaction partners. Most importantly, our experimental design emphasizes the tension between aligned interests in one’s network and an explicitly imposed social norm of truth-telling. People in a network can be dishonest without being held fully accountable for their lies. In spreading false information, people can “hide” behind the lies of others so that the recipient of a lie cannot be sure about who is responsible for that lie. Furthermore, people embedded in networks can contribute to spreading false information without necessarily lying about the information they receive. The experiment that we discuss in the next section captures these aspects of a naturally occurring social network.

Adding to these benefits, the experiment we design also helps us to better understand the mechanisms that may drive the effectivity of verification, such as the psychological cost that individuals experience when telling lies or the reputational cost they perceive when identified as liars^13^. We interrogate these mechanisms through experimental manipulations that change the presence and type of verification to test which of these channels may enhance the effect of verification. Our findings can help inform the designs of useful interventions and policies to prevent the spread and amplification of lies on social networks in real-world settings, where people are surrounded by others who are similar to them, when sharing information^25–27^.

### Experimental design

**Figure 1:**
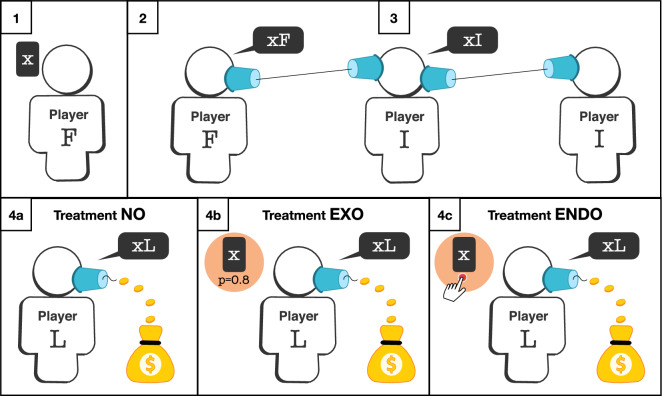
Experimental game and main treatment variations. (1) The *web of lies* game begins with player *F* who draws a card with a hidden number, *x*, between 1 and 30; (2) player *F* then reports a number, *xF*, to the intermediate player, *I*; (3) player *I* observes *xF* and reports a number, *xI*, to the last player, *L*; (4) player *L* observes *xI* and reports a number, *xL*, to the experimenter. The goal of the game is to report $xL=x$ to the experimenter, but players are paid according to *xL*, which gives them an incentive to over-report. Experimental treatments manipulate the last player’s ability to observe the true value of *x* before making the final report; (4a) treatment no is the baseline, where player *L* does not receive any information on *x*; (4b) treatment exo is a condition with exogenous verification, where player *L* has an $80\%$ chance of learning the value of *x* before sending his report of *xL*; (4c) treatment endo is a condition with endogenous verification, where player *L* can click on a button to observe the value of *x* before sending his report, *xL*.

We design a one-shot sequential game, which we call the *web of lies* game (see Fig. 1), where three players are assigned to different positions in a linear communication network: first, *F*, intermediate, *I*, and last, *L*. At the beginning of the game, player *F* chooses a card from a $12 \times 12$ grid, which reveals an integer, *x*, between 1 and 30 written on the card. The number *x* is observed only by *F* and is referred to as the *hidden number*. Player *F* then sends a number, *xF*, also between 1 and 30, to player *I*, reporting on *x*. Player *I* observes *xF*, but not *x*, and reports a number, *xI*, under the same conditions to player *L*. Finally, player *L* observes *xI*, but not *x* or *xF*, and reports the final number, *xL*, this time to the experimenter.

Each of the three players earn 5 cents multiplied by the number reported by the last player, e.g., if $$xL=20$$, then each of the players make $1.00. This means that the monetary compensation linearly increases in the number reported by the last player and that the monetary incentives of people in the communication network are perfectly aligned. It is common knowledge that the report that players send need not match the number they observed. This creates the possibility of over-reporting, i.e., the possibility of lying, and the payoff is highest when $$xL=30$$. However, all players are told that the goal of the game is for them to send reports so that the last player can report the same number that was drawn by the first player. That is, *xL* should be equal to *x*. This rule, which is restated on the screen where players make their decisions, institutes a norm of truth-telling.

We choose a distribution of hidden numbers where any integer between 1 and 30 has a positive probability of being drawn by player *F*, which ensures that no reports are obvious lies. As there are more cards with smaller numbers, the probability is higher for smaller numbers to be drawn, which is known to all (see Supplementary Information Sect. 7 for the full instrument). In sum, the truth is costly, as far as the monetary incentives of the players are concerned, and lies may be suspected based on the size of the reported number but are never evident without verification. From a normative perspective, however, lies come at a cost, as players have been informed how they *should* play the game.

In this setting, to evaluate the role of verification on lying, we designed three experimental treatments that manipulate the last player’s ability to observe the value of the hidden number, *x*, before making the final report (see Fig. 1). That is, we focus our implementation of verification on player *L*, whose report is the only payoff-relevant report for the group. The first treatment is a baseline with *no verification* of the hidden number (no). That is, the game is as described above, where player *L* makes the final report after observing *xI* and has no additional information on *x* (see Fig. 14a). The second treatment introduces *exogenous verification* (exo), such that the last player has an $80\%$ chance of learning *x* after receiving *xI* but before making his or her report (see Fig. 14b). In each communication network, there is an 80% chance that we reveal the hidden number; thus, 80% of the groups are expected to see the hidden number in this condition; deviations are a result of random chance. Substantively, we implement a fact checker as a centralized, external source for the veracity of the information. Finally, the third treatment is one with *endogenous verification* (endo) where the last player, after receiving *xI*, can verify *x* by clicking on a button (see Fig. 14c). This means that, unlike in exo, player *L* in endo can choose to avoid information by not clicking on the button. There is no monetary cost of verifying and observing *x* in the verification treatments. Importantly, verification allows player *L* to identify whether the number she received, *xI*, is a lie or not but not who the liar is. It may be that player *F*, player *I*, or both lied.

In addition, we elicited beliefs (incentivized) in both verification treatments by asking players *F* and *I*, after making their report, whether they believed the last player had verified the number, that is, if *L* had been shown (in exo) or had clicked on a button to see (in endo) the true value of *x* (see the instructions in Supplementary Information Sect. 7). By such means, we can evaluate how the anticipation of verification impacted players’ behavior and their incentive to lie.

The experiment was programmed in oTree^28^ so that all interactions between participants took place through a web interface. We recruited participants from Amazon Mechanical Turk, which has recently gained prominence in collecting behavioral data online^29,30^. See the criteria for participation in the Methods section and descriptive statistics of the participants in Supplementary Information Tables Sect. S1 and Sect. S2. In total, 2177 people participated in nine experimental conditions, with approximately 80 groups per condition. On average, the duration of the experiment was 10 min, and participants earned approximately $2.00, resulting in a hourly wage of $12.0. The study was approved by the institutional review board at NYU Abu Dhabi (#063-2018). The standard measures of anonymity and non-deception were used, i.e., participants in the experiment received accurate and comprehensive information about the experimental condition they participated in. All experiments were performed in accordance with relevant guidelines and regulations and by obtaining online informed consent from participants.

## Results

In our analysis, we make multiple comparisons between the outcomes of the different experimental treatments using two-sided t-tests and report *p* values in the text. We report marginally significant results at the 0.1 level as well, and for each test, we report the corresponding *p* values, emphasizing which results are significant at the usual level of 0.05 and which are marginally significant at the level of 0.1. See the results of power calculations in Supplementary Information Sect. 2 justifying this choice and suggesting that the study is underpowered to detect small effects but reasonably powered to detect medium-sized effects^31^.

To preview the structure of our analysis and main results, first, we quantify the effect of verification on the spread of lies at the group level and find that verification has limited effectivity in the prevention of lies. To establish these findings, we use the three treatments already described, no, endo, and exo. Second, we try to enhance its effectiveness in additional experimental treatments using two strategies: using two additional treatments, we increase the psychological cost of lying by introducing passive players whose payoffs are reduced with false reports. Moreover, using three additional treatments, we increase the reputational cost of lying by making evident who lies. Of these two strategies, only the latter works.

### The main effect of verification on the spread of lies

To evaluate how effective verification is in reducing the spread of false reports, we focus on two key measures: the likelihood of lying and the size of the lies told. We begin by analyzing group-level outcomes and then turn to the behavior of participants in each position in the communication chain.

**Figure 2:**
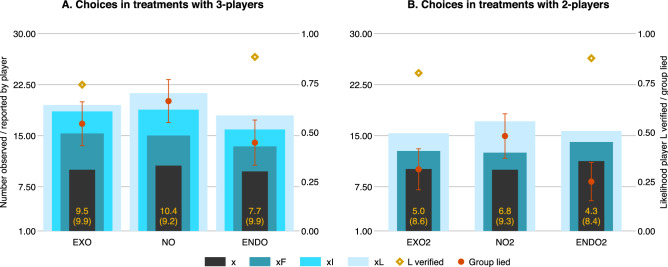
Main decisions made by players and treatments in the 3-person games (Panel **A**) and in the 2-person games (Panel **B**). The bars indicate the numbers observed and reported by each player in the game (left vertical axis). *x* is the hidden number, *xF* is the number reported by player *F*, and the difference between the two bars is the magnitude of the lies told by first players. *xI* is the number reported by the intermediate player *I*, and the difference with the *xF* bar is the magnitude of *I*’s lie. *xL* is the number reported by the last player, *L*, where the difference between *xL* and *xI* is the magnitude of *L*’s lies, and the difference between *xL* and *x* is the magnitude of the lie at the group level. The mean and SD of the magnitude of the group lies are at the bottom of each bar. The circle dots indicate the share of groups that lie in each treatment, error bars are $\pm \,1$ SE, and the diamond dots indicate the share of players *L* who verify the number (right vertical axis).

At the group level, a lie is reported if the final report is different from the hidden number, $xL \ne x$, and the size of the lie told is the magnitude of that difference, $xL-x$ (see bars in Fig. 2A). We compare the share of lying groups and the average size of lies told in each treatment with verification to the treatment with no verification. We find that only endogenous verification is effective in preventing the spread of lies, as groups in endo lie 22 percentage points less often ($p = 0.004$) and tell smaller lies, 7.7 versus 10.4 ($p = 0.084$, i.e., marginally significant), than those in no. In contrast, compared to groups in no, groups in exo lie at the same rate ($p = 0.118$) and by an indistinguishable amount (10.4 vs. 9.5, respectively, $p = 0.576$).

Importantly, this moderate effect on preventing lies in the treatments with verification is not due to low levels of actual verification of the truth (see diamonds in Fig. 2A). Verification was randomly assigned to a large share of groups in exo and was chosen by an even larger share in endo ($75\%<89\%$, $p = 0.020$). Even though verification in endo is a choice and is not experimentally imposed, there is little evidence of information avoidance. In Supplementary Information Sect. 3, we provide evidence that differences between endo and exo when compared to no are mostly driven by mechanisms *unrelated* to differences in verification. Therefore, to assess what is driving the observed effectivity of endogenous verification (and the lack thereof of exogenous verification), we analyze the way reports and lies are spread by participants in the different positions in the network.

First, we consider the hidden number, *x*, which is the value the first player is asked to report on. Because *x* is a result of a draw in each group and is not randomized, we compare the distribution of hidden numbers across treatments to rule out differences in outcomes being based on the conditions groups start in instead of being based on our experimental manipulations (i.e., failed randomization). We find that the average *x*s drawn, 10.6 in no, 10.0 in exo, and 9.7 in endo, are not significantly different across treatments (see first bars in Fig. 2A as well as Supplementary Information Sect. 4), indicating that groups in all three treatments analyzed here start in equivalent conditions.

Second, we analyze the reports made by player *F* in the network. The average reports by player *F* were 15.0, 15.3, and 13.4 in no, exo and endo, respectively. Player *F* lies by reporting a different number than the hidden number that he or she drew ($xF - x \ne 0$), and the size of the lie is the magnitude of that difference (see the gap between the first and second bars in Fig. 2A). Neither the probability of lying nor the size of the lies are affected by the anticipation of exogenous ($p=0.788$ and $p=0.976$) or endogenous verification ($p=0.407$ and $p=0.468$) when compared to the baseline condition of no verification. This suggests that knowing the last player could identify whether the number reported to him or her is false does not impact the lying behavior of first players.

Third, we consider the reports made by players in the intermediate position, *I*. The average reports by player *I* were 18.7, 18.2, and 15.8 in no, exo and endo, respectively. Player *I* lies by reporting a different number than the one he or she received from player *F* ($xI - xF \ne 0$), and the size of the lie is the magnitude of that difference (see the gap between the second and third bars in Fig. 2A). Note that player *I* may report a lie by repeating the report he or she received from player *F* if player *F* lied. Compared to the baseline, there are no significant differences in the magnitude of the lies told by player *I* in endo ($p=0.244$) or in exo ($p=0.732$). However, the probability that player *I* lies is significantly smaller in the endogenous verification condition ($p=0.046$). When we analyze how the reports from the intermediate player differ from the hidden number (see the gap between the first and third bars in Fig. 2A), we find that there is a significantly lower share of false reports reaching the final player in endo than in no ($p = 0.045$). Moreover, smaller numbers are reported by the intermediate player $15.9 < 18.8$ ($p=0.058$, i.e., marginally significant). In contrast, no statistically significant differences are found between the exogenous verification and no verification conditions in either case. That is, while in both verification conditions, a downward shift is observed in lies and reports, the impact of exogenous verification cannot be distinguished from zero.

Taken together, the evidence so far suggests that player *L* receives different messages on average when endogenous verification is implemented than when no verification is implemented, while no such difference exists between the exogenous verification and no verification conditions. This is driven by differences in the behavior of the intermediate player, possibly due to the proximity to the last player, who is the one doing the verification. Differences in proximity may indicate players being wary of being found out as liars. We test this empirically by examining players’ beliefs and anticipating that those who believe that they will be verified lie less. A total of 78% of *F* and *I* players in endo believed that player *L* would verify and observe the true value of *x*. When comparing the reports of those who believed that the last player would check the hidden number to those who believed the opposite, the differences are not statistically significant considering each role, *F* and *I*, separately ($p > 0.100$). When we pool our data for all non-final players, we find that those who believed that the last player would verify reported significantly smaller numbers than those who believed no verification would occur ($p = 0.060$, i.e., marginally significant).

We finally turn to the role that the final players have in stalling the spread of lies when verifying the truth. A large share of *L* players repeat the report they received from player *I* ($xL = xI$). However, when the message received is identified as false, the spread of lies by repeating the report received is significantly lower than in the baseline, both in terms of the probability of telling a lie and the size of the lie told (see Tables S4–S7 in Supplementary Information Sect. 5). Moreover, in endo, where player *L* had agency in identifying the truth, he or she is more likely to correct a lie and submit a smaller final report than final players in exo, where observing the true value of *x* is not a choice (see the gap between the third and fourth bars in Fig. 2A; also note the negative interaction terms in Tables S6 and S7 in Supplementary Information Sect. 5, with *p* values of $p = 0.100$ and $p = 0.093$, respectively, i.e., indicating marginally significant results).

Our analyses reveal that endogenous verification is a more effective intervention than exogenous verification, which is also more effective than no verification. We identify two avenues via which this occurs. On the one hand, endogenous verification reduces the number of lies told by those who anticipate that their lies will be identified. On the other hand, it appears to trigger the motivation among those who have the agency to verify the truth to correct a lie and report the truth. However, our data reveal that groups still report lies in endo and that the size of lies told is only moderately lower than that in no. Drawing from the literature on lying behavior and the effects of transparency, there are multiple mechanisms behind truth telling. The two most prominently discussed are the *psychological costs of lying* and *concern for reputation*^13^. We explore six new treatments that aim to elevate these costs to increase the effectiveness of verification when verification is endogenous.

### Increasing the psychological cost of lying to enhance the impact of verification

First, we address how increasing the psychological cost of lying may enhance the efficacy of verification. For this, we designed two additional treatments using endo, where we add one or two passive players, creating victims of lies, whose payoffs decrease as a function of the lies told by player *L*. We label these treatments vctm when there is a single passive player and vctms when there are two passive players. Victims make no decisions, and their payoffs are 5 cents $\times (2x - xL)$. This means that victims earn the same as the active players when the last report is truthful ($xL = x$), but their payoffs are negatively affected by lies. This simple point is made clear to participants by stating that truth-telling results in an equal payoff for everyone. Unlike in endo, in vctm and vctms, lying by reporting a number different from the hidden number hurts others, which is expected to increase the psychological cost of lying for final players. Thus, we evaluated whether the presence of negative externalities decreases the share of groups that lie or reduces the size of the lies that groups tell relative to endo.

We find that having a victim (vctm) does not affect the rate at which the last players verified the truth ($p=0.935$) relative to the victimless case of endo. This holds true even when the number of victims who are hurt is increased to two (vctms), making the size of the group that is hurt by lies equal to the size of the group that benefits from lies, disregarding player *L* ($p=0.657$). Moreover, the reports made by players in all three positions and the frequency and amount of lying were not different from endo in vctm or vctms (see Fig. S4, Supplementary Information Sect. 6). In sum, we find that increasing the cost of lying by introducing negative externalities does not seem to increase the efficacy of verification. These findings are consistent with the previous findings regarding ignorance towards third-party externalities^32^ and triggering a rational mode of thinking, which encourages cheating^33^. The findings, however, differ from those of Abeler and colleagues^13^. What drives these discrepant results is a concern for future work and extends beyond the scope of this experiment.

### Increasing the reputational cost of lying to enhance the impact of verification

Next, we test whether the effectiveness of verification can be enhanced by increasing the reputational costs of lying for non-final players. We expect that players would want to avoid being seen as liars by their group members. We find indicative evidence of this conjecture when we observe that the expectation of verification has a stronger effect on players who believe that their reports will be verified and that players positioned closer to the one verifying lie less. To enhance the salience of the reputational mechanism, we design three additional experimental treatments where not only the lie but also the liar can be identified through verification. We achieve this by removing the intermediate player *I* from the communication chain, which means that we now analyze a 2-player *web of lies* game.

In this setting, we test the role of verification, as we did before, by comparing a treatment with no verification (no2) to two forms of verification: exogenous verification (exo2) and endogenous verification (endo2). In this 2-player web of lies game, the first player *F* observes *x* and reports a number to the last player *L*. Therefore, if *F* tells a lie, he or she risks being identified as a liar and cannot hide behind another group member who may have also lied. This is true even in no2, where *L* has no certainty that *F* is a liar but is aware of the distribution from which *x* was drawn (note that in the 3-player game, the likelihood of a lie is also identifiable but the source of the lie is not). In other words, diffusing responsibility for a lie is not possible^34^. As a corollary, reputational concerns are high and virtually equal across experimental conditions.

Eliminating the intermediate player, and thus the possibility of hiding behind others, results in first players telling fewer and smaller lies than those in the same position in the main treatments with 3-players, in no2 ($2.6 < 4.8, p=0.058$), exo2 ($2.6 < 5.2, p=0.034$) and endo2 ($2.8 < 3.8, p=0.393$—note that although the effect is present, it is not significant in endo2, probably due to floor effects). Moreover, participants in the position of player *F* do not differ in their behavior across treatments in the 2-player game either (see Fig. 2B). This provides an ideal test of the effect of verification, given that player *L* observes statistically indistinguishable messages (and lies) when making his or her report. Our findings show that at the group level, removing the intermediate player leads to less frequent and smaller lies than in the 3-player treatments (no2 $p=0.016$, exo2 $p=0.002$ and endo2 $p=0.020$).

Consistent with our previous findings, the effect in the conditions with verification (exo2 and endo2) is not driven by differences in the rates of verification, which are not different across communication chain lengths (see diamonds in Fig. 2B). In addition, evaluating the effect of verification, we find that when compared to no2, both exo2 and endo2 are effective in reducing the share of lying groups: $p = 0.033$ and $p = 0.002$, respectively (see circles in Fig. 2B). However, the same effect is only marginally significant in reducing the size of lies for endo2 ($p = 0.078$) and is not significant for exo2 ($p = 0.209$). This supports our finding suggesting that the choice to verify *x*, rather than simply the observation of its true value, strongly drives truth-telling.

Our findings from the treatments with the increased exposure of the liar provide new insights into existing experimental evidence showing that higher scrutiny has little impact on dishonesty. Although it has been suggested that transparency policies are not always a remedy against dishonesty, we find that being exposed as a liar to a group member, as in our case, has a significantly different effect than being exposed to other third parties, which proves ineffective^35^, or to the experimenter, which can even be counterproductive^24^.

Finally, we conduct an additional treatment to interrogate whether increasing both reputational and psychological costs has a compound effect. Namely, we introduce a passive victim to the endogenous verification treatment in the 2-player game (vctm2) and find that the effectivity of verification does not increase ($p =0.992$). Thus, in addition to increasing reputational costs by eliminating the intermediate player, there is no additional effect on verification when introducing negative externalities from lying.

We conclude that reputational mechanisms are key to promoting the effectiveness of verification, while enhancing the psychological cost of lying does not appear to be effective.

### Limitations

While this work has some key advantages, it is not without limitations. The very strength of our experimental approach also results in some weaknesses. Importantly, in real-world settings, centralized fact-checkers not only tag lies but also carry the reputation of the institution and the true or presumed ideologies of that institution. In our experiment, we cannot speak to how participants’ behaviors would change as a function of this additional signal. Modification of the experiment to incorporate these nuances is possible but is left to future work. Moreover, in this experiment, all participants’ information about the possible state of the world was the same, i.e., nobody was more or less informed or had varying levels of expertise to probe what might be true, which is not always the case in reality. This latter point may mask heterogeneity about the willingness to spread lies by those who are more knowledgeable or informed in the substantive domain concerning the piece of information they receive, their group identity notwithstanding. Similar to the abovementioned point, this is another important avenue for future research.

## Discussion

Our work highlights some major limitations of verification interventions for preventing the spread of false information. The spread of lies on networks that are organized around shared interests, such as echo-chambers, is difficult to prevent. Even when an explicit norm of truth-telling is instituted or when lies can affect third parties outside the group, verification is only moderately effective. This is so because simply revealing lies is insufficient to stop people from benefiting themselves and their group members, which may be a legitimizing force behind spreading lies in such settings. However, when people have agency and choose to verify the truthfulness of information, it is this seeking behavior that enhances their adherence to the norm of telling the truth^36^. This is evident in the comparison of endogenous and exogenous verification. Individuals who make the choice to verify the truth about the information they receive spread fewer lies when observed but are also less likely to receive false information from their network. As such, a policy focused on exposing truth may have a weaker effect than one in which individuals are equipped to uncover for themselves whether the information they receive is truthful.

In large social networks, simply introducing verification may not be effective^37^, for it may be easier to hide behind others, and detrimental outcomes can result without clear culprits. This became evident in our comparison between the simplest case of 2-person and 3-person networks. By having someone to whom the responsibility for lying could be transferred, individuals were significantly more willing to lie. This points to the idea that in complex networks, beyond the 3-person case of our study, the likelihood and magnitude of lies is expected to increase because individuals are able to hide behind their many connections when considering linear communication chains. Naturally, the larger the size of the lie the more likely that it is identifiable. However, the experimental evidence has shown that individuals avoid lying maximally to minimize their chances of being caught^13,38^. In fact, in our setting the level of maximal lying (i.e., groups reporting $x=30$) is not higher than $33\%$. Larger networks, however, also have more complex network structure, as naturally occurring networks often contain cycles. In our design we maintained a simple line structure for it is ideal to address our research question. However, different network structures could promote disclosure, if the person sharing information also takes into account that the same information may have been shared by another, mutual, contact before. In this case, the focal actor might consider that the information they spread could be contrasted to previous reports, and might refrain from exaggerating what they hear to protect their reputation. To fully characterize how a more complex network structure may alter behavior in these ways is an important avenue of research that we aim to explore in future work.

These results taken together indicate that being effective in countering the spread of false information is a necessary uphill battle: the culture must be changed so that it values truth, and the source or sources where lies originate must be tracked so that liars can be identified. Although this can be challenging, especially in a “post-truth” society, our work provides insights into how to tackle this problem. As evidenced by our results, sharing common interests strengthens groups and may facilitate the spread of lies, even when imposing harm on others. However, it is this same drive from caring about one’s connections that could be used to strengthen the efficacy of verification. Specifically, we observe that the potential of being exposed as a liar to those one is connected to significantly increases adherence to the truth-telling norm. Therefore, verification strategies work best when not only lies but liars are identified, as people aim to maintain their good reputation before those they care about.

## Methods

### Inclusion criteria

The experimental sessions took place between 3rd and 21st August 2018. Participants were recruited through Amazon Mechanical Turk (MTurk). We restricted participation to subjects who were 18 years or older and with a geographic location in the United States, as specified on their MTurk account and by their IP address.

Participants needed to have at least a 95% approval rating on prior MTurk projects and at least 100 prior MTurk projects approved—which is associated with careful attention to instructions in online surveys in general. In addition, after reading the instructions of the study, participants were asked a set of comprehension check questions. Only those who answered all questions correctly were able to finish the study. They were allowed two attempts, and the instructions were available to them at all times. In case they failed, they were directed to an end-of-survey message and did not take part in the experimental game. In other words, we only included participants in our analysis who gave a clear indication of having understood experimental procedures and the compensation structure of the game.

### Compensation

All participants who completed the study earned $1.00 for participating. In addition to the fixed payment, participants earned a bonus dependent on two factors. The first was the final report made, which gave them 5 cents $$\times xL$$ as a bonus. In the treatments with victims, it was possible for the bonus to be negative for the passive players. In such cases, the bonus payment was set to zero, i.e., the participants could not lose any portion of their participation payment. This was clearly explained to participants as part of the instructions for the game. The second source of variation in payments was based on participants’ answers about their beliefs regarding how others played the game. Namely, each participant answered a set of questions about his or her group members’ behavior; one of the questions was randomly chosen for payment, and if it was answered correctly, it would result in a bonus of 20 cents. The average earnings of active participants are included in Tables S1 and S2 in Supplementary Information Sect. 1 and are broken down by experimental condition.

## Supplementary information

Supplementary material 1 (pdf 0 KB)
