# Author Correction: Web of lies: a tool for determining the limits of verification in preventing the spread of false information on networks

**DOI:** 10.1038/s41598-021-94888-w

**Published:** 2021-07-28

**Authors:** Kinga Makovi, Manuel Muñoz-Herrera

**Affiliations:** Social Science Division, New York University, Abu Dhabi, UAE

Correction to: *Scientific Reports* 10.1038/s41598-021-82844-7, published online 15 February 2021

The Acknowledgements section in the original version of this Article was incomplete.

“We are grateful for the constructive comments received from Andrzej Baranski, Sanjeev Goyal, Agne Kajakaite, Byungkyu Lee, Georgia Michailidou, Rebecca Morton, Nikos Nikiforakis, Daniele Nosenzo, Wojtek Przepiorka, Ernesto Reuben, and Marie Claire Villeval as well as the participants of the 2019 Winter Experimental Social Sciences Institute at NYU Abu Dhabi, International Meeting on Experimental and Behavioral Social Sciences at the University of Utrecht and the participants of the Networks and Time speaker series at Columbia University. We also thank the anonymous reviewer for generous comments that helped improve the paper.”

now reads:

“We are grateful for the constructive comments received from Andrzej Baranski, Sanjeev Goyal, Agne Kajakaite, Byungkyu Lee, Georgia Michailidou, Rebecca Morton, Nikos Nikiforakis, Daniele Nosenzo, Wojtek Przepiorka, Ernesto Reuben, and Marie Claire Villeval as well as the participants of the 2019 Winter Experimental Social Sciences Institute at NYU Abu Dhabi, International Meeting on Experimental and Behavioral Social Sciences at the University of Utrecht and the participants of the Networks and Time speaker series at Columbia University. We also thank the anonymous reviewer for generous comments that helped improve the paper. This work was supported by the NYUAD Center for Interacting Urban Networks (CITIES), funded by Tamkeen under the NYUAD Research Institute Award CG001 and by the Swiss Re Institute under the Quantum Cities™ initiative.”

The original Article has been corrected.

