# Supplementary material for: Web of lies: a tool for determining the limits of verification in preventing the spread of false information on networks
Source: Sci Rep. 2021 Feb 15;11:3845. doi: 10.1038/s41598-021-82844-7 (PMC7884844; doi:10.1038/s41598-021-82844-7)
Supplement: Supplementary file 1 — Supplementary material 1 (pdf 0 KB) [file 41598_2021_82844_MOESM1_ESM.pdf]

Supplementary Information
Web of lies: a tool for determining the limits of verification in preventing the spread of false information on networks

Kinga Makovi[1,*+] and Manuel Munñoz-Herrera[1+]

[1]New York University, Social Science Division, Abu Dhabi, UAE
[*]km2537@nyu.edu
[+]Both authors contributed equally to this work and are listed alphabetically

ABSTRACT

The spread of false information on social networks has garnered ample scientific and popular attention. To counteract this spread, verification of the truthfulness of information has been proposed as a key intervention. Using a novel behavioral experiment with over 2000 participants, we analyze participants' willingness to spread false information in a network. All participants in the network have aligned incentives, making lying attractive, countering an explicit norm of truth-telling that we imposed. We investigate how verifying the truth, endogenously or exogenously, impacts the choices to lie or to adhere to the norm of truth-telling, compared to a setting without the possibility of verification. The three key take-aways are: (i) verification is only moderately effective in reducing the spread of lies; (ii) its effectivity is contingent on the agency of people to seek truth, and (iii) on the exposure of liars, and not only the lies told. These suggest that verification is not a blanket solution. In order to enhance its effectivity, it should be combined with fostering a culture of truth-seeking and with information on who spreads lies.

1 Descriptive statistics

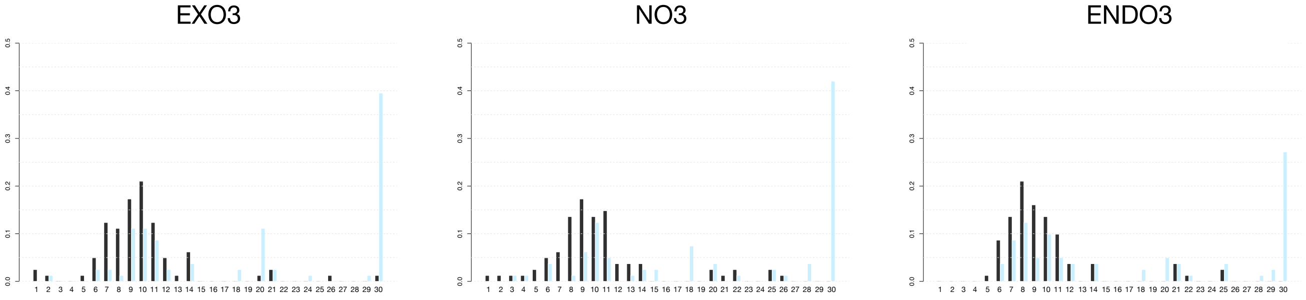

Figure S1. Destribution of the hidden number (x) and final report (xL) in the 3-player treatments

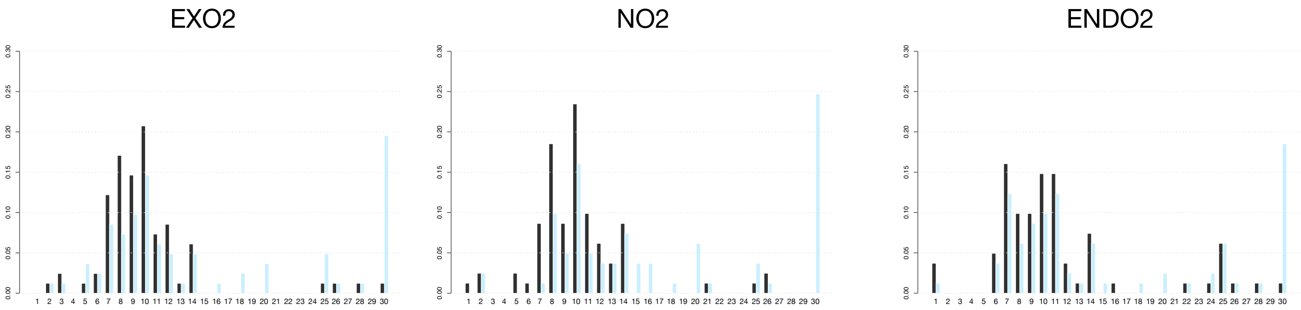

Figure S2. Destribution of the hidden number (x) and final report (xL) in the 2-player treatments

Table S1. Descriptive statistics for treatments in the 3-player games.

Variable	NO	EXO	ENDO	VCTM	VCTMS
N of groups	80	83	80	83	77
N of participants	240	249	240	332	385
Gender (%)					
Female	48.3	51.0	47.5	50.3	50.4
Male	51.3	47.8	52.1	49.4	48.8
Other	0.4	1.2	0.4	0.3	0.8
Race (%)					
Non-Hispanic White	78.8	84.7	80.4	79.2	80.8
Other than non-Hispanic White	21.2	15.3	19.6	20.8	19.2
Education (%)					
High school or less	6.3	10.0	10.8	9.9	7.5
Some college	30.8	32.1	33.3	26.8	30.1
College diploma	47.1	44.6	42.1	48.5	45.7
More than college degree	15.8	13.3	13.8	14.8	16.6
Income (%)					
Less than $10,000	13.8	15.3	13.8	16.0	13.8
$10,000 - $19,999	10.0	6.4	12.9	9.0	9.1
$20,000 - $29,999	12.1	14.5	11.2	11.1	13.5
$30,000 - $39,999	14.6	14.1	14.6	11.1	11.7
$40,000 - $49,999	12.5	9.2	11.7	10.5	13.5
$50,000 - $69,999	17.9	18.1	16.2	20.8	18.7
More than $70,000	19.2	22.5	19.6	21.4	19.7
Age (mean years)	34.8	35.3	34.1	33.8	34.5
Compensation (mean $ of active participants)	$2.04	$1.98	$1.88	$1.96	$1.91

Table S2. Descriptive statistics for treatments in the 2-player games.

Variable	NO2	EXO2	ENDO2	VCTM2
N of groups	81	82	81	81
N of participants	162	164	162	243
Gender (%)				
Female	51.9	54.9	48.8	50.2
Male	48.1	45.1	51.2	49.4
Other	0.0	0.0	0.0	0.4
Race (%)				
Non-Hispanic White	82.1	75.0	84.0	78.2
Other than non-Hispanic White	17.9	25.0	16.0	21.8
Education (%)				
High school or less	7.4	7.3	7.4	11.1
Some college	28.4	32.3	30.9	26.3
College diploma	49.4	47.0	46.3	45.0
More than college degree	14.8	13.4	15.4	17.7
Income (%)				
Less than $10,000	9.9	15.9	11.7	14.4
$10,000 - $19,999	13.0	9.8	4.9	9.5
$20,000 - $29,999	11.1	11.6	16.1	14.4
$30,000 - $39,999	16.1	14.0	14.2	14.4
$40,000 - $49,999	11.7	11.0	11.1	8.6
$50,000 - $69,999	17.3	18.3	20.4	18.1
More than $70,000	21.0	19.5	21.6	20.6
Age (mean years)	35.0	34.7	35.0	35.0
Compensation (mean $ of active participants)	$1.85	$1.76	$1.78	$1.76

2 Power calculation

(a) (b)

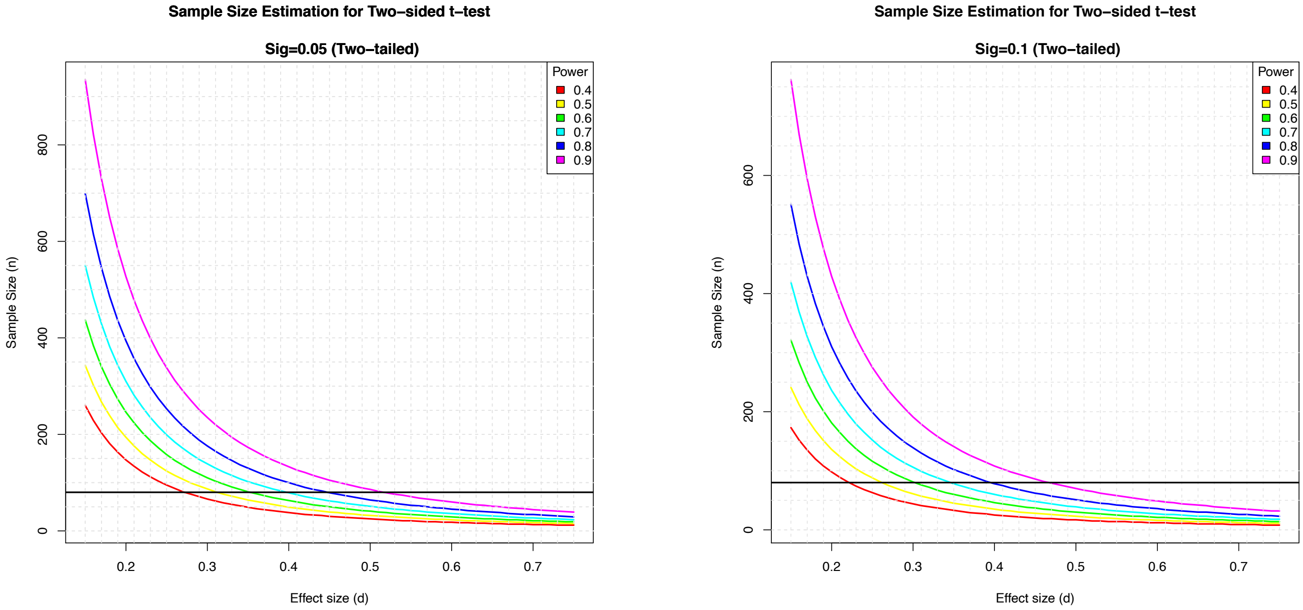

Figure S3. Results of power-calculation

Here we provide the results of a power analysis following Cohen, conducted in R using the pwr package[1]. We anticipate small to moderate effect as the share of individuals lying in the Fischbacher and Folmi-Heusi dice rolling game is 60%[2], giving room to the effect of being observed in this ranfe. Based on Cohen's recommendation, this corresponds to an effect size of d between 0.2 and 0.5. Given our sample size of 80 per experimental condition, represented on the y-axis in Fig. S3a, with a confidence level of 0.05, we are under-powered to detect small effects. Specifically, we are powered at the level of 0.241 for small effects ($d = 0.2$), while we are powered at the level of 0.882 for medium-sized effects ($d = 0.5$). When decreasing our level of certainty to 0.10, see Fig. S3b, the level of power corresponding to small and medium-sized effects are 0.352 and 0.934, respectively. Summing it up, with the samples at our disposal we are weakly powered to detect small effects, while we may detect medium-sized effects.

3 Testing for alternative explanation: differences in rates of verification

Naturally, unlike EXO, verification in ENDO is a consequence of individual choices, and thus, it is not experimentally imposed on final players. We anticipated this issue, and calibrated the probability of showing the hidden number in EXO based on a small pre-test of the experiment, and sat this probability at 80%. In each group, independently of one another, we showed the hidden number to the final player with this probability. Given this independence, we in fact ended up with only 75% of final players seeing the hidden number in EXO. Additionally, we ended up with a higher rate of verification in ENDO than the pre-test, specifically, 89% of final players verified.

To address this gap, we reweighted the observations in EXO so that the weighted observations yield the same rate of verification as observed in ENDO, and then compared again NO and EXO. We find a slight decrease in the probability that a group lies (p = 0.061, marginally significant), but no differences in the size of lies that groups tell (p = 0.429). This suggests that differences in rates of verification are driven by an alternative mechanism to differential rates of verification when compared to no verification.

4 Comparison of the hidden numbers drawn by experimental condition

Table S3. Comparing differences in means in the hidden number across conditions.

	EXO	ENDO	NO2	EXO2	ENDO2	VCTM	VCTMS
NO	0.569	0.430	0.764	0.659	0.343	0.522	0.692
EXO		0.822	0.769	0.903	0.138	0.214	0.337
ENDO			0.598	0.731	0.092	0.140	0.240
NO2				0.871	0.209	0.326	0.480
EXO2					0.175	0.272	0.407
ENDO2						0.703	0.570
VCTM							0.825

5 Regression analyses

Table S4. Modeling the probability of lying at the group level comparing NO to the verification conditions EXO and ENDO using logistic regression.

	Estimate	Std. Error	Pr(> $|z|$)
Intercept	-0.773	0.349	0.027*
Verification	-0.062	0.429	0.886
Received a lie	4.487	1.071	<0.001***
Verification × Received a lie	-3.037	1.134	0.007**

Table S5. Modeling the final report at the group level comparing NO to the verification conditions EXO and ENDO using OLS regression.

	Estimate	Std. Error	Pr(> $|z|$)
Intercept	15.342	1.391	<0.001***
Verification	-0.118	1.704	0.945
Received a lie	10.372	1.920	<0.001***
Verification × Received a lie	-4.473	2.438	0.068.

Table S6. Modeling the probability of lying at the group level comparing EXO to ENDO using logistic regression.

	Estimate	Std. Error	Pr(> $|z|$)
Intercept	-1.056	0.411	0.010*
Endogenous	0.363	0.518	0.484
Received a lie	2.112	0.581	<0.001***
Endogenous × Received a lie	-1.265	0.769	0.100

Table S7. Modeling the final report at the group level comparing EXO to ENDO using OLS regression.

	Estimate	Std. Error	Pr(> $|z|$)
Intercept	14.419	1.599	<0.001***
Endogenous	1.358	2.078	0.515
Received a lie	8.516	2.262	<0.001***
Endogenous × Received a lie	-5.332	3.151	0.093

6 Supplementary figures

Figure S4 reports the main decisions for treatments with passive victims, compared to treatments with endogenous verification, both for the 3-person and the 2-person games.

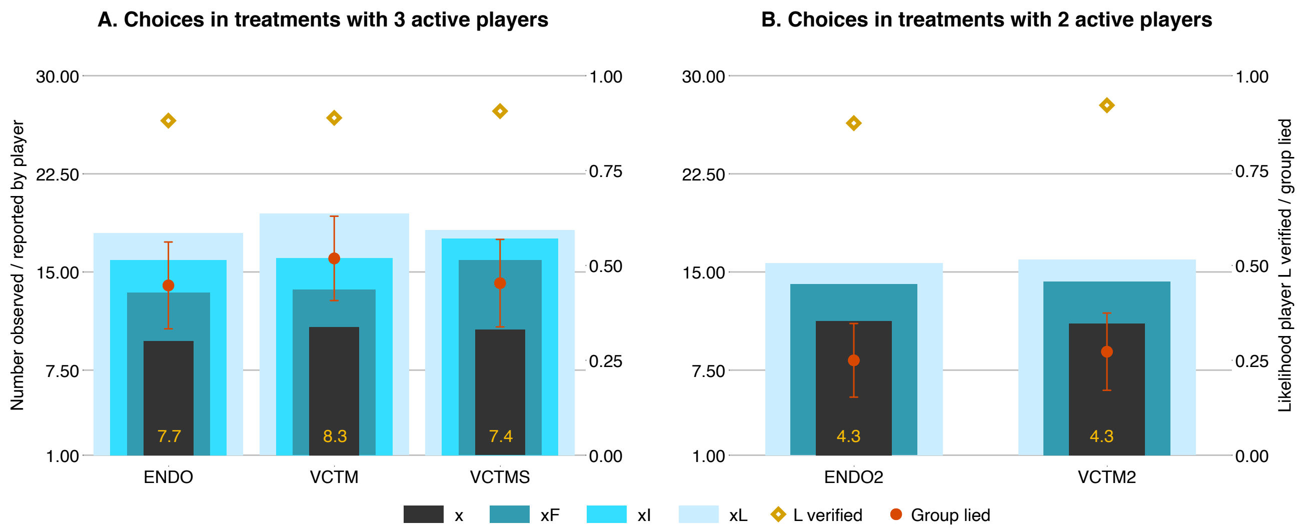

Figure S4. Main decisions by players and treatments in the 3-person games (Panel A) and in the 2-person games (Panel B) with victims. The bars indicate the numbers observed and reported by each player in the game (left vertical axis). x is the hidden number, xF is the number reported by player F and the difference between the two bars is the magnitude of the lies told by first players; xI is the number reported by the intermediate player I and the difference with the xF bar is the magnitude of I's lie; and xL is the number reported by the last player L, where the difference between xL and xI is the magnitude of L's lies, while the difference between xL and x is the magnitude of the lie at the group level. The mean and SD of the magnitude of the group lies are at the bottom of each bar. The circle dots indicate the likelihood that a group lies in each treatment, error bars are ± 1 SE; and the diamond dots the likelihood that player L verifies (right vertical axis).

7 Instructions

PAGE 1

Welcome to this study. You will play a simple game with other MTurk workers, the details of which will be explained to you in the following screens. You will read the instructions for the tasks and be asked a few comprehension questions to see if you understand the instructions. You can only participate if you answer all of these questions correctly, so your full attention is needed for the duration of the study.

Your participation will last between 5 and 10 minutes.

For participating in the study, you will earn a show-up payment of $1. You can earn up to $2.7 in these tasks in addition to your show-up payment. How to earn this money is explained to you in the instructions.

Please, click on the button to begin your participation in this study.

PAGE 2

Sociodemographic Questionnaire
Please answer the questions below.

1. What is your gender?
    - Male
    - Female
    - Other
2. What is your Ethnicity?
    - Hispanic / Latino / Latina
    - Not Hispanic / Latino / Latina
3. What is your Race?
    - White
    - Black / African American
    - Asian
    - American Indian / Alaskan Native
    - Middle Eastern / North African
    - Native Hawaiian / Pacific Islander
    - Other
4. What is your age?
5. What is your highest completed level of education?
    - Less than high school
    - High school diploma or equivalent (e.g., GED)
    - Some college
    - College diploma
    - Masters degree

- Professional post-secondary degree or doctoral degree (e.g., JD, MD, PhD etc.)

6. Which state do you live in?
7. What was your yearly personal income in 2017 (include salary, interests, returns on investments)?
    - Less than $10,000
    - $10,000 – $19,999
    - $20,000 – $29,999
    - $30,000 – $39,999
    - $40,000 – $49,999
    - $50,000 – $59,999
    - $60,000 – $69,999
    - $70,000 – $79,999
    - $80,000 – $99,999
    - $100,000 – $119,999
    - $120,000 – $149,999
    - $150,000 – $199,999
    - $200,000–
    - I do not wish to report my income.

PAGE 3

Below we present the instructions for treatment NO *with 3 active players. In the following subsections we illustrate differences in instructions for treatments* EXO*,* ENDO *and* VCTM*. The instructions for the 2-person games are as the ones we present, without the information for the intermediate player.*

Please read the instructions carefully. After you finish reading them you will be asked some comprehension questions to verify that you understand the instructions. You can only participate in the study once you have answered all of the questions correctly. If you did not get all questions right, you will have the opportunity to view the instructions again, and revise your answers one more time. **If you fail twice you will not be able to participate in this study, and you will not get paid.** As a consequence, your full attention is required for the duration of the study.

Instructions. You will earn money based on the decisions you and others make in this game explained below. **This payment is over and above the payment of** $1 **dollar that you will be paid for this HIT.**

The first task you will complete is explained here, and you will receive instructions for any additional tasks later. In the first task, you will play a game in a group with two other participants who are also workers on Amazon Mechanical Turk.

You will be labeled as P1, P2, or P3, which will indicate the order in which you play. Depending on your label, you will be the one drawing the **hidden number**, you will send a message to the next player, or submit a **final message**.

The objective of the game is to send messages so that P3 would report in the **final message** the **hidden number** drawn by P1.

The task of the different participants is illustrated in detail in the image, and is described below:

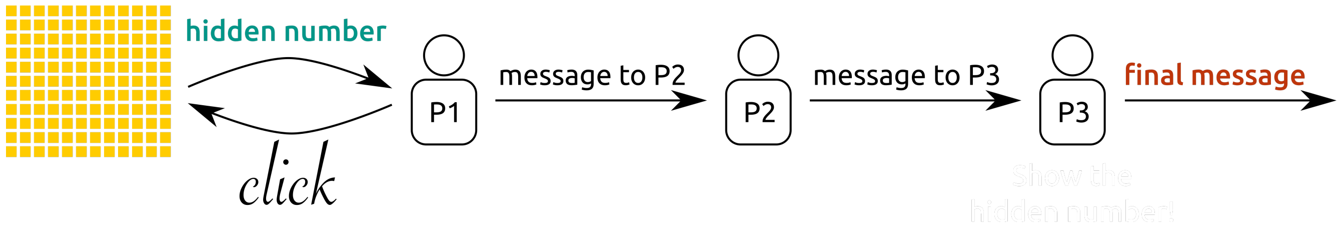

Figure S5. Decisions in the study

1. The game starts with P1 who clicks on one of the 144 cards arranged in a 12 by 12 grid appearing on the screen which can be seen on the right of the image below. Each card has a number on it between 1 and 30, and clicking on a card reveals the number, which will be the **hidden number** in the game you play.

   The frequency of each number on the cards is illustrated on the left of the image at the bottom of these instructions. Note that each of the possible numbers appear on at least one card. However, there are some numbers that are more frequent than others. For example, there are four cards with the number 5 on them, there are sixteen cards with the number 7 on them, or thirty cards with the number 10 on them - which is the most frequent number -, while there is only one card with, say, the number 20 on it.

2. The **hidden number** is revealed to P1 when he/she clicks on a card. Then, P1 will send a message reporting on the **hidden number** to P2.

3. P2 will see the number P1 reported in his/her message, but will not see the **hidden number** drawn by P1. Then, P2 will send a message to P3 reporting on the **hidden number**.

4. P3 will see the number P2 reported in his/her message. P3 will not see the **hidden number** that was drawn by P1. Then, P3 sends a **final message** reporting on the **hidden number**.

Earnings

Note that the earnings in this task are additional to your payment of $1 dollar that you will be paid for this HIT regardless of the choices you and others make in this game.

All participants get paid according to the number P3 reports in the **final message**.

P1, P2 and P3 each earns 5 cents times the number P3 reports in the **final message**. For example, if the number reported by P3 is 16, then P1, P2 and P3 will earn 5*16 = 80 cents.

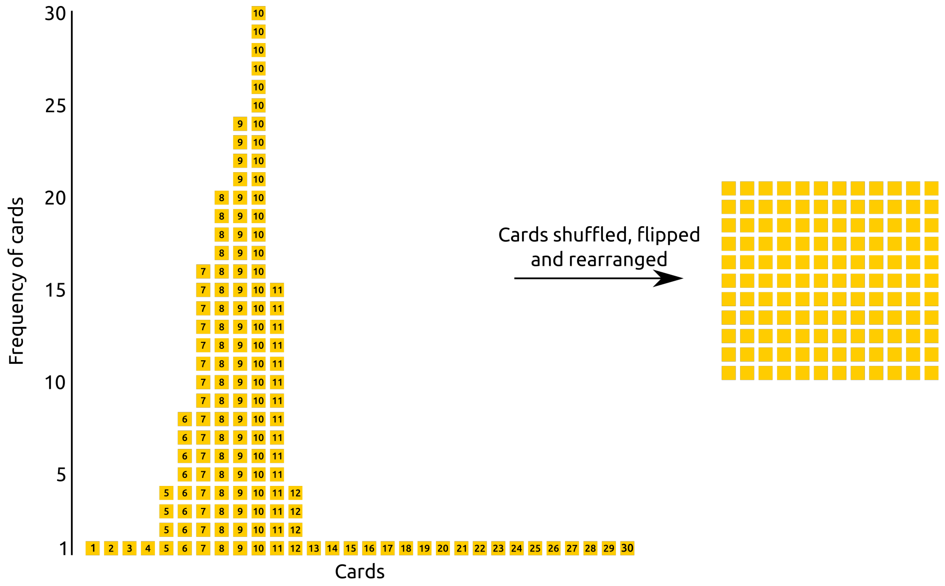

Figure S6. Distribution of the hidden number

These instructions will be displayed at the bottom of your screen if you click on a “Show Instructions” button.

If you have read the instructions **carefully**, press the button below. You can only participate in the study once you have answered correctly **all** of the questions testing your comprehension of the instructions, on the following screen. If you do not get all questions right, you will have an opportunity to review the instructions again, and revise your answers one more time. **If you fail twice, you will not be able to participate in this study, and you will not get paid**. As a consequence, your full attention is required for the duration of the study.

PAGE 3 (other treatments)

Treatment EXO

The instructions for treatment EXO differ from NO in point #4, as follows:

4. P3 will see the number P2 reported in his/her message. P3 will also see the **hidden number** that was drawn by P1 with 80% chance. This means that with 20% chance P3 will not see the **hidden number**. Then, P3 sends a **final message** reporting on the **hidden number**.

Treatment ENDO

The instructions for treatment ENDO differ from NO in point #4, as follows:

4. P3 will see the number P2 reported in his/her message. P3 can also check the **hidden number** that was drawn by P1 by clicking on a button displayed on his/her screen. P3 will not see the **hidden number** if he/she does not click on the “Show the hidden number” button. Then, P3 sends a **final message** reporting on the **hidden number**.

Treatment VCTM

The instructions for treatment VCTM differ from NO in point #4, has an addition point #5 and a different description of earnings, as follows:

4. P3 will see the number P2 reported in his/her message. P3 can also check the **hidden number** that was drawn by P1 by clicking on a button displayed on his/her screen. P3 will not see the **hidden number** if he/she does not click on the “Show the hidden number” button. Then, P3 sends a **final message** reporting on the **hidden number**.
5. P4 does not receive or send any messages in this task, he/she is a passive player.

Earnings

All four participants get paid according to the number P3 reports in the **final message**, but the payment will be calculated differently for P4 than for the other three players (P1, P2, and P3).

P1, P2 and P3 each earns 5 cents times the number P3 reports in the **final message**. For example, if the number reported by P3 is 16, then P1, P2 and P3 will earn 5*16 = 80 cents.

P4, on the other hand, will be paid 5 cents times the difference between twice the **hidden number** and the **final message** – which is (2***hidden number** - **final message***5 cents. For example, if the **hidden number** is 10 and P3 reported 16 in the **final message**, then P4 will earn (2*10-16)*5 = (20-16)*5 = 4*4 = 20 cents.

If the resulting earnings for P4 would be negative, he/she earns 0 cents in this task.

Note that if the **hidden number** and the**final message** are the same, all players earn the same amount.

PAGE 4

Comprehension Questionnaire

Please answer these questions carefully. You can only participate in the study once you have answered all of them correctly. If you answer one or more incorrectly, you will get a chance to review your answers again for a second time. If you have any doubts, you can click on the "Show Instructions" button below and the instructions will be displayed.

1. **P1 will draw a hidden number between 1 and 30 from the cards displayed in the instructions. Which of these numbers is the most likely to be drawn as the hidden number?**
    - 10
    - 4
    - 25
    - 18
    - 20
2. **P1 will send a message reporting on the hidden number to P2. What information will P2 receive?**
    - P2 will see the number reported by P1, and with 80% chance will also see the hidden number
    - P2 will see the number reported by P1 and will see the hidden number if he/she clicks on a button so that it shows on the screen
    - P2 will see the number reported by P1 but not the hidden number
    - P2 will not see the number reported by P1, only the hidden number
    - P2 will not see any messages, he/she is a passive player
3. **P2 will send a message reporting on the hidden number to P3. What information will P3 receive?**
    - P3 will see the number reported by P2, and with 80% chance will also see the hidden number
    - P3 will see the number reported by P2 and will see the hidden number if he/she clicks on a button so that it shows on the screen
    - P3 will see the number reported by P2 but not the hidden number
    - P3 will not see the number reported by P2, only the hidden number
    - P3 will not see any messages, he/she is a passive player
4. **Suppose the hidden number is 12 and P3 reports 12 in the final message. How many cents do participants P1, P2 or P3 earn aside from their show-up payment?**
    - 24×5 = 120 cents

- $6 \times 5 = 30$ cents
- $12 \times 5 = 60$ cents
- $18 \times 5 = 90$ cents
- It cannot be determined from this information alone

5. **Suppose the hidden number is 12 and P3 reports 18 in the final message. How many cents do participants P1, P2 or P3 earn aside from their show-up payment?**

    - $24 \times 5 = 120$ cents
    - $6 \times 5 = 30$ cents
    - $12 \times 5 = 60$ cents
    - $18 \times 5 = 90$ cents
    - It cannot be determined from this information alone

Belief Questionnaire

At the end of the study, before receiving information about the final report or their earnings, participants responded to a series of belief elicitation questions as follows:

Instructions

In this task you can earn **extra money** by answering a few questions. We will ask you to report how you think **other participants** played in this game you just participated in.

You will be asked a few questions in this task and **one of them** will be randomly selected for payment.

You will earn **20 cents** depending on the accuracy of your answer for the randomly selected question.

Player 1

- This question is about the choice made by another participant **in your group**. You can earn **20 cents** if your report is 3 units above or below the choice made by the other participant if this question is selected for payment. For example, if the participant reported 5, and your answer is between 2 and 8, you will earn 20 cents. Otherwise, you will not earn money in this task.

  The message you sent to P2 reporting on the **hidden number** was [*report from P1 was displayed here*]. What number do you think P2 sent to P3 after seeing your report?

- EXO: This question is about the information observed by participant **P3 in your group**. If your answer is accurate, you will earn **20 cents** in case this question is selected for payment.

  In addition to seeing P2's report, there is an 80% chance that P3 would see the **hidden number** drawn. Do you think P3 observed the **hidden number**?

- ENDO: This question is about the choice made by participant **P3 in your group**. If your answer is accurate, you will earn **20 cents** in case this question is selected for payment

  In addition to seeing P2's report, P3 could click on a button to see the **hidden number** drawn. Do you think P3 clicked on the button and observed the **hidden number**?

- This question is about the information observed by another participant **in your group**. You can earn **20 cents** if your report is 3 units above or below the information observed by the other participant. For example, if the participant observed 5 and your answer is between 2 and 8, you will earn 20 cents. Otherwise, you will not earn money in this task.

  You said that the message P2 sent to P3 reporting on the **hidden number** was [*belief reported by P1 to the first question was displayed here*]. What number do you think P3 sent in the **final message**?

Player 2

- This question is about the information observed by another participant **in your group**. You can earn **20 cents** if your report is 3 units above or below the information observed by the other participant. For example, if the participant observed 5 and your answer is between 2 and 8, you will earn 20 cents. Otherwise, you will not earn money in this task.

  The message P1 sent to you reporting on the **hidden number** was [*report from P1 was displayed here*]. What number do you think P1 drew as the **hidden number** before sending you his report?

- EXO: This question is about the choice made by participant **P3 in your group**. If your answer is accurate, you will earn **20 cents** in case this question is selected for payment

  In addition to seeing your report, there is an 80% chance that P3 would see the **hidden number** drawn. Do you think P3 observed the **hidden number**?

- ENDO: This question is about the information observed by participant **P3 in your group**. If your answer is accurate, you will earn **20 cents** in case this question is selected for payment.

  In addition to seeing your report, P3 could click on a button to see the **hidden number** drawn. Do you think P3 clicked on the button and observed the **hidden number**?

- This question is about the choice made by another participant **in your group**. You can earn **20 cents** if your report is 3 units above or below the choice made by the other participant if this question is selected for payment. For example, if the participant reported 5, and your answer is between 2 and 8, you will earn 20 cents. Otherwise, you will not earn money in this task.

  The message you sent to P3 reporting on the **hidden number** was [*belief reported by P2 to the first question was displayed here*]. What number do you think P3 sent in the **final message**?

Player 3

- This question is about the information observed by another participant **in your group**. You can earn **20 cents** if your report is 3 units above or below the information observed by the other participant. For example, if the participant observed 5 and your answer is between 2 and 8, you will earn 20 cents. Otherwise, you will not earn money in this task.

  The message you received from P2 reporting on the **hidden number** was [*report from P2 was displayed here*]. What number do you think P2 received from P1 before sending you this message?

- **Only if P3 did not verify the true value of the hidden number**. This question is about the information observed by another participant **in your group**. You can earn **20 cents** if your report is 3 units above or below the information observed by the other participant. For example, if the participant observed 5 and your answer is between 2 and 8, you will earn 20 cents. Otherwise, you will not earn money in this task.

  You said the message P1 sent to P2 reporting on the **hidden number** was [*belief reported by P3 to the first question was displayed here*]. What number do you think P1 drew as the **hidden number** before sending his report to P2?

8 Decision Screens

Treatment NO

In Figure S7 we display the decision screens for all three players (P1, P2 and P3) in treatment NO.

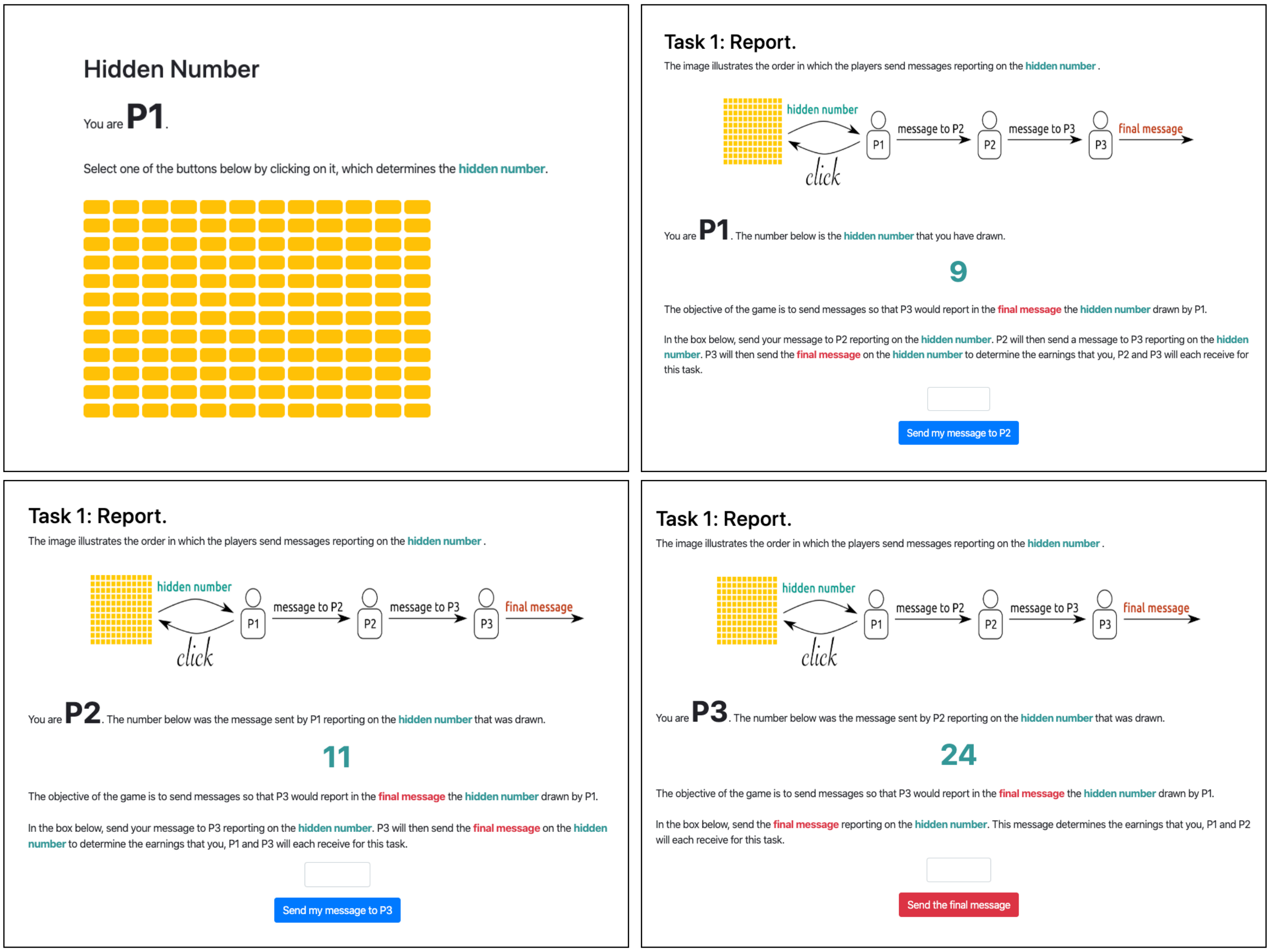

Figure S7. Decision screens in NO. In NO, the *web of lies* game begins with (Upper-left) P1 choosing a card from a 12×12 grid by click on it, each card with an integer between 1 and 30, (Upper-right) P1 then reports a number to P2, (Lower-left) P2 observes the number sent by P1 and reports a number to P3, (Lower-right) P3 observes the number sent by P2 and sends a report to the experimenter.

Treatment EXO

In Figure S8 we display the decision screens for the last player (P3) in treatment EXO.

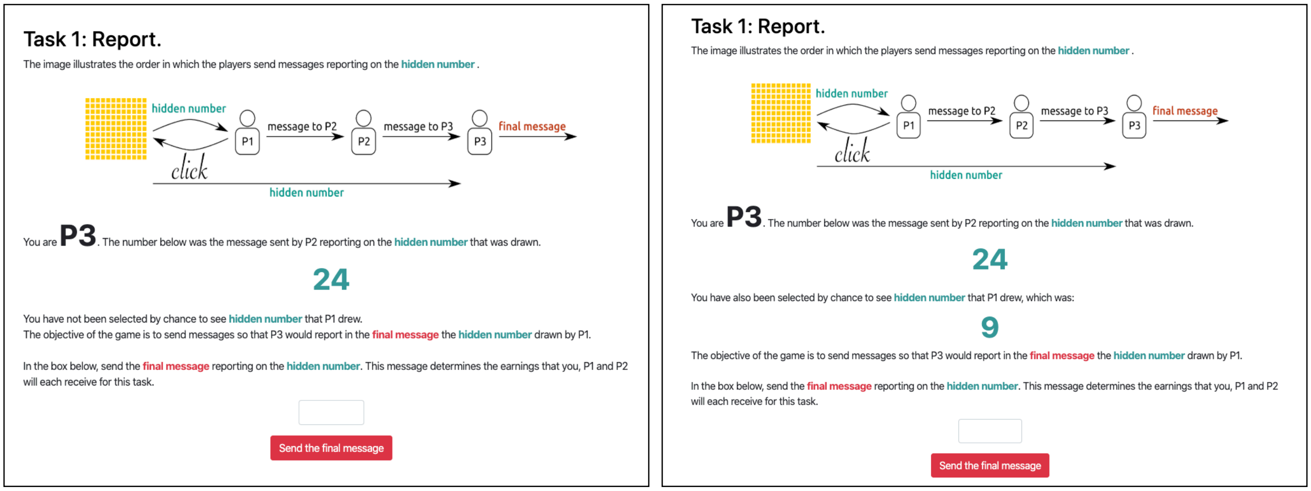

Figure S8. Decision screens in EXO. In EXO P1 and P2 make decisions with identical information as in NO. (Left) P3 has the same info in EXO as in NO, only observes the report from P2, with 20% chance. (Right) P3 observes both the report from P2 and the hidden number with 80% chance.

Treatment ENDO

In Figure S9 we display the decision screens for the last player (P3) in treatment ENDO.

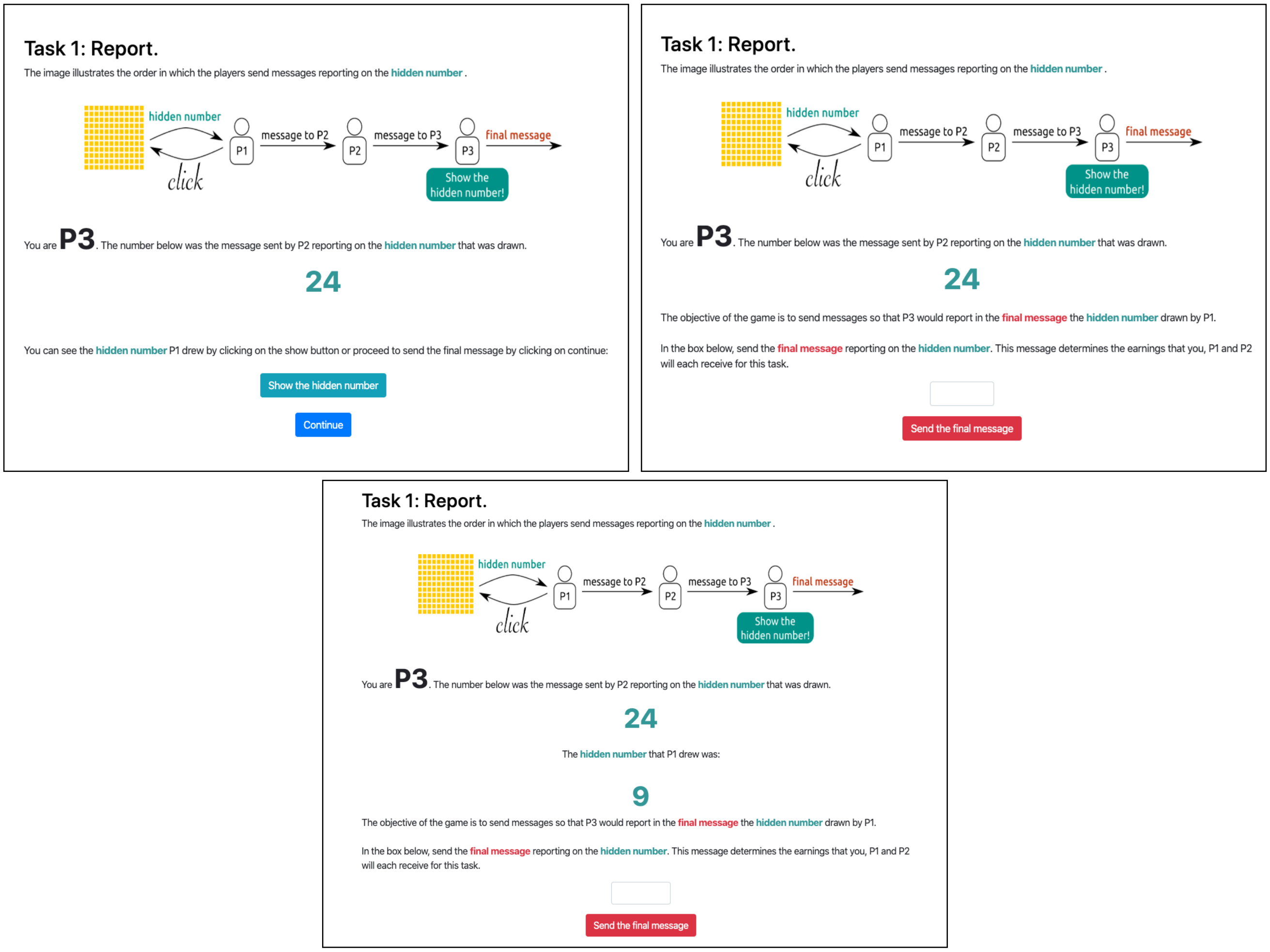

Figure S9. Decision screens in ENDO. In ENDO P1 and P2 make decisions with identical information as in NO. (Upper-Left) P3 has the option to click on the "Show the hidden number" button to verify it or to click on the "Continue" button and avoid seeing the hidden number. (Upper-Right) If P3 avoids verification, he only observes the report from P2. (Bottom) If P3 decides to verify, he observes both the report from P2 and the hidden number.

References

1. Cohen, J. *Statistical Power Analysis for the Behavioral Sciences, Second Edition* (Routledge, New York, 1988).

2. Fischbacher, U. & Follmi-Heusi, F. Lies in disguise: An experimental study on cheating. *J. Eur. Econ. Assoc.* **11**, 525–547 (2013).
